# Supplementary material for: Association of Alternative Dietary Patterns with Osteoporosis and Fracture Risk in Older People: A Scoping Review
Source: Nutrients. 2023 Oct 3;15(19):4255. doi: 10.3390/nu15194255 (PMC10574803; doi:10.3390/nu15194255)
Supplement: Supplementary file 1 [file nutrients-15-04255-s001.zip › nutrients-2616353-supplementary.pdf]

Ovid MEDLINE(R) Search Strategy

#	**Search term**
1	exp Aged/
2	aged.ti,ab.
3	elderly.ti,ab.
4	((older or senior*) adj2 (people or individual* or person* or m?n or wom?n or adult*)).ti,ab.
5	1 or 2 or 3 or 4
6	Dietary Approaches To Stop Hypertension/
7	(Diet* adj3 (pattern* or variant* or score*)).ti,ab.
8	(Nordic diet* adj2 (score* or assess* or scale* or index or indices)).ti,ab.
9	(Baltic Sea diet* adj2 (score* or assess* or scale* or index or indices)).ti,ab.
10	(Mediterranean diet* adj2 score*).ti,ab.
11	(Alternative Health Eating Index or AHEI).ti,ab.
12	(Health Eating Index or HEI).ti,ab.
13	(Alternative Mediterranean Diet or AMED).ti,ab.
14	(DASH diet* adj2 (score* or assess* or scale* or index or indices)).ti,ab.
15	(Portfolio diet* adj2 (score* or assess* or scale* or index or indices)).ti,ab.
16	(NOMA diet* adj2 (score* or assess* or scale* or index or indices)).ti,ab.
17	(Chinese diet* adj2 (score* or assess* or scale* or index or indices)).ti,ab.
18	(Japanese diet* adj2 (score* or assess* or scale* or index or indices)).ti,ab.
19	(Korean diet* adj2 (score* or assess* or scale* or index or indices)).ti,ab.
20	(Asia* diet* adj2 (score* or assess* or scale* or index or indices)).ti,ab.
21	(Latin* diet* adj2 (score* or assess* or scale* or index or indices)).ti,ab.
22	(Polish diet* adj2 (score* or assess* or scale* or index or indices)).ti,ab.
23	or/6-22
24	fractures, bone/ or exp femoral fractures/ or osteoporotic fractures/ or rib fractures/ or shoulder fractures/ or spinal fractures/ or exp ulna fractures/ or fractures, multiple/
25	exp Osteoporosis/
26	Bone density/
27	((bone* or hip* or femoral or femur* or osteoporo* or rib* or shoulder* or spinal or spine* or ulna or wrist* or multiple or fragility or vertebr* or lumbar) adj2 fracture*).ti,ab.
28	or/24-27
29	5 and 23 and 28
30	limit 29 to (english language and yr="2000 -Current")
